# Association between caloric adequacy and short-term clinical outcomes in critically ill patients using a weight-based equation: Secondary analysis of a cluster-randomized controlled trial

**DOI:** 10.3389/fnut.2022.902986

**Published:** 2022-09-02

**Authors:** Cheng Lv, Xingwei Jiang, Yi Long, Zirui Liu, Jiajia Lin, Cuili Wu, Xianghong Ye, Ruiling Ye, Yuxiu Liu, Man Liu, Yang Liu, Wensong Chen, Lin Gao, Zhihui Tong, Lu Ke, Zhengying Jiang, Weiqin Li

**Affiliations:** ^1^Department of Critical Care Medicine, Jinling Hospital, Medical School of Nanjing University, Nanjing, China; ^2^Department of Critical Care Medicine, Jinling Hospital, Medical School of Southeast University, Nanjing, China; ^3^Department of Critical Care Medicine, Chongqing University Cancer Hospital, Chongqing, China; ^4^Department of General Surgery, Jinling Hospital, Medical School of Nanjing University, Nanjing, China; ^5^Department of Biostatistics, School of Public Health, Nanjing Medical University, Nanjing, China; ^6^National Institute of Healthcare Data Science, Nanjing University, Nanjing, China

**Keywords:** energy intake, mortality, hypocaloric feeding, resting energy expenditure, critical illness

## Abstract

**Background:**

There is controversy over the optimal energy delivery in intensive care units (ICUs). In this study, we aimed to evaluate the association between different caloric adequacy assessed by a weight-based equation and short-term clinical outcomes in a cohort of critically ill patients.

**Methods:**

This is a secondary analysis of a cluster-randomized controlled trial (*N* = 2,772). The energy requirement was estimated as 25 kcal/kg of body weight. The study subjects were divided into three groups according to their caloric adequacy as calculated by the mean energy delivered from days 3 to 7 of enrollment divided by the estimated energy requirements: (1) received < 70% of energy requirement (hypocaloric), (2) received 70–100% of energy requirement (normocaloric), and (3) received > 100% of energy requirement (hypercaloric). Cox proportional hazards models were used to analyze the association between caloric adequacy and 28-day mortality and time to discharge alive from the ICU.

**Results:**

A total of 1,694 patients were included. Compared with normocaloric feeding, hypocaloric feeding significantly increased the risk of 28-day mortality (hazard ratio [*HR*] = 1.590, 95% confidence interval [*CI*]: 1.162–2.176, *p* = 0.004), while hypercaloric feeding did not. After controlling for potential confounders, the association remained valid (adjusted *HR* = 1.596, 95% *CI*: 1.150–2.215, *p* = 0.005). The caloric adequacy was not associated with time to discharge alive from the ICU in the unadjusted and the adjusted models.

**Conclusion:**

Energy delivery below 70% of the estimated energy requirement during days 3–7 of critical illness is associated with 28-day mortality.

**Clinical trial registration:**

[https://www.isrctn.com/ISRCTN12233792], identifier [ISRCTN12233792].

## Introduction

Nutrition therapy is essential in the management of critically ill patients, as they are vulnerable to energy/protein deficits due to severe catabolism and inadequate intake, which may result in increased infectious complications and prolonged intensive care unit (ICU) stay (1, 2). However, the optimal energy delivery for critical illness remains controversial, particularly during the early phase of ICU admission (3, 4). Notably, both insufficient and excessive calorie intakes are reported to be associated with poor outcomes (5–8).

The European Society for Clinical Nutrition and Metabolism (ESPEN) guidelines (9) recommend progressively providing energy to avoid overfeeding and cover at least 70% of the needs within 3–7 days. The recommendation rests on a large retrospective study including 1,171 critically ill patients, which revealed that the optimal energy intake appeared to be between 70 and 100% of the energy requirement (8). Since they used indirect calorimetry (IC) for energy requirement measurement, the generalizability of this finding in patients without IC was unknown (10). Given that IC is not readily available in all settings, especially in medium and low-income countries, weight-based equations are commonly applied in the absence of IC measurement (9, 11–14).

In the present study, we classified caloric adequacy using the three-category system (<70% as hypocaloric, 70–100% as normocaloric, and >100% as hypercaloric) based on a weight-based equation (25 kcal/kg/day). We aimed to investigate the association between caloric adequacy and short-term clinical outcomes in a cohort of critically ill patients from a large trial.

## Materials and methods

### Study design and patients

This study is a *post hoc* analysis of a multicenter, cluster-randomized, controlled trial assessing the impact of an evidence-based nutrition guideline in critically ill patients (15). The trial was approved by the local hospital ethics committees of all the participating ICUs and registered at the ISRCTN registry (ISRCTN12233792). Briefly, a total of 2,772 patients with an expected ICU stay longer than 7 days were enrolled within 24 h of ICU admission from 90 ICUs between March 26, 2018 and July 4, 2019. Our analysis is conducted on a subset of the participants. Inclusion criteria were (1) an ICU stay longer than 3 days and (2) having at least one evaluable nutrition day. We excluded patients who received PN during the first 48 h in light of the mainstream guidelines (9, 16). Additionally, patients who received an oral diet were also excluded because the calories from the oral diet cannot be calculated. Evaluable nutrition days were defined as the days when patients only received artificial nutrition (EN or PN) from days 3 to 7 after enrollment.

### Energy intake and patients grouping

Energy intake was calculated only on the evaluable days because the calories from the oral diet were impossible to calculate precisely. The caloric adequacy was determined by comparing the mean actual mean daily energy delivery from days 3 to 7 of enrollment with the target energy requirement as a percentage. The target energy requirements were estimated based on a simple weight-based equation (25 kcal/kg of actual body weight on admission day).

All eligible patients were divided into three groups according to their caloric adequacy: (1) received < 70% of energy requirement (hypocaloric group), (2) received 70–100% of energy requirement (normocaloric group), and (3) received > 100% of energy requirement (hypercaloric group).

### Outcomes and definitions

The coprimary outcomes are 28-day mortality and time to discharge alive from the ICU. The secondary outcomes include the length of ICU stay and new receipt of organ support therapy within the first 7 days after enrollment. Time to discharge alive from the ICU was only considered in survivors and censored at 28 days after enrollment. New receipt of organ support therapy was defined as a requirement of organ support therapy (mechanical ventilation, renal replacement therapy, and vasoactive agents) not applied at enrollment.

### Data collection

All data were extracted from the electronic database, such as de-identified data on patient characteristics, daily nutritional therapy, and utilization of organ support. The baseline characteristics included age, gender, weight, body mass index (BMI), modified Nutrition Risk in the Critically Ill (mNUTRIC) score (17), site before ICU admission, comorbidities, Acute Gastrointestinal Injury (AGI) score (18), and severity scores, such as Acute Physiology and Chronic Health Evaluation II (APACHE II) score (19) and sequential organ failure assessment (SOFA) score (20) at enrollment. Nutrition therapy variables included daily nutritional route (oral, enteral, and parenteral), enteral or parenteral formulas, protein supplements, hours with feed, and the feed-volume delivered in milliliters. In addition, energy provided by dextrose-containing intravenous fluids was included in the calculation of total energy intake. Nutritional intake was recorded within the first 7 days after enrollment or until discharge from the ICU or death, whichever occurred first.

### Statistical analyses

The analysis was performed using SPSS version 25 (SPSS Inc., Chicago, IL, United States) and R software version 4.1.0. The normality of continuous variables was examined by the Kolmogorov–Smirnov test. Continuous data were presented as mean ± standard deviation (SD) or median (interquartile range, IQR). Categorical data were presented as numbers and percentages. Differences in baseline and nutritional characteristics among the three energy delivery categories were compared using the one-way analysis of variance (ANOVA) or the Kruskal–Wallis tests for continuous data and the chi-square test for categorical data. A Bonferroni correction was applied for the *post hoc* pairwise comparisons. The Kaplan–Meier methods were used to display survival curves for time to discharge alive from the ICU within 28 days after enrollment and 28-day mortality. Noncross- and cross-survival curves were compared by the log-rank test and two-stage test (21), respectively.

Cox proportional hazards models were used to analyze the effect of caloric adequacy on 28-day mortality and time to discharge alive from the ICU. The covariates, such as age, gender, BMI, APACHE II score, SOFA score, number of comorbidities, mNUTIRC score, the number of evaluable nutrition days, initiation of enteral nutrition within 48 h, mean parental nutrition intake from days 3 to 7, and AGI score were entered separately in the multivariable model. The relationships between caloric adequacy and the two coprimary outcomes were tested in the unadjusted and the adjusted models. Based on unadjusted analyses, variables of clinical significance or with *p* < 0.1 were considered covariates and included in the adjusted model 1.

The multicollinearity between potential confounding factors was evaluated in the multivariable model by assessing the variance inflation factor (VIF) (22, 23). The fit of the Cox model was assessed using Cox-Snell residuals. The proportional hazards assumption was tested by plotting Schoenfeld residuals. A two-tailed *p*-value of <0.05 was considered significant.

## Results

### Patients characteristics

**FIGURE 1:**
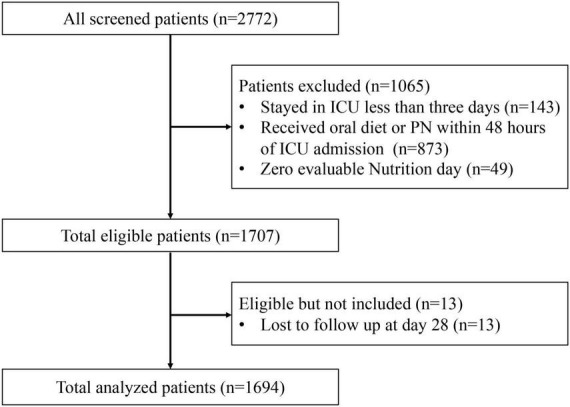
Eligibility screening.

All the 2,772 trial participants were screened for potential inclusion, and 1,694 patients were included in the current analysis (Figure 1). A total of 823 patients were assigned to the hypocaloric group, 571 to the normocaloric group, and 300 to the hypercaloric group. Table 1 describes the baseline characteristics and clinical outcomes of the study population among three different categories of energy adequacy. The majority of patients had a mild gastrointestinal dysfunction with AGI grade I (*n* = 1,251, 73.8%) and were admitted to the emergency department (*n* = 706, 41.7%). The overall 28-day mortality was 13.1% (222/1694), and the median length of ICU stay was 16 days (interquartile range: 9–28 days). The variables of age, gender, BMI, and SOFA score were different among the three groups, whereas others were similar among the groups.

**TABLE 1:** Baseline characteristics and clinical outcomes in the study population and among three different categories of energy adequacy.

	Total (*n* = 1694)	Hypocaloric (*n* = 823)	Normocaloric (*n* = 571)	Hypercaloric (*n* = 300)	*P*-value
**Groups**					
Intervention group	1003 (59.3)	467 (56.7)	348 (60.9)	188 (62.6)	0.118
Control group	691 (40.7)	356 (43.3)	223 (39.1)	112 (37.3)	
Age	63 (49–74)	61 (47–73)	64 (51–76)	63 (50–75)	0.016
Male	1129 (66.6)	599 (72.8)	361 (63.2)	169 (56.3)	<0.001
BMI	22.7 (20.8–24.5)	23.3 (21.6–25.3)	22.5 (20.8–24.2)	21.3 (19.0–23.0)	<0.001
APACHE II	18 (14–23)	18 (14–23)	18 (13–22)	18 (13–23)	0.128
SOFA	7 (5–10)	8 (5–11)	7 (5–9)	7 (5–10)	<0.001
Number of co-morbidities	2 (1–3)	2 (1–3)	2 (1–3)	2 (1–3)	0.326
**Co-morbidity**					
Hypertension	761 (44.9)	373 (45.3)	264 (46.2)	124 (41.3)	0.358
Diabetes	315 (18.5)	168 (20.4)	109 (19.0)	38 (12.6)	0.012
Coronary disease	295 (17.4)	144 (17.4)	107 (18.7)	44 (14.6)	0.297
Stroke	272 (16.0)	135 (16.4)	92 (16.1)	45 (15.0)	0.860
mNUTRIC score	4 (3–6)	4 (3–6)	4 (3–5)	4 (3–6)	0.238
**Nutrition risk**					
Low risk (mNUTRIC < 5)	948 (56.0)	445 (54.0)	334 (58.4)	169 (56.3)	0.287
High risk (mNUTRIC ≥ 5)	746 (44.0)	378 (46.0)	237 (41.6)	131 (43.7)	
**AGI score**					
AGI I	1251 (73.8)	552 (67.1)	462 (81.0)	237 (79.0)	<0.001
AGI II	325 (19.2)	192 (23.3)	83 (14.5)	50 (16.6)	
AGI III	94 (5.5)	63 (7.6)	20 (3.5)	11 (3.6)	
AGI IV	24 (1.4)	16 (1.9)	6 (1.0)	2 (0.6)	
**Site before ICU admission**					
Emergency department	706 (41.7)	326 (39.6)	258 (45.2)	122 (40.7)	0.047
Surgical department	391 (23.1)	212 (25.7)	117 (20.5)	62 (20.7)	
Medical department	305 (18)	152 (18.5)	104 (18.0)	50 (16.6)	
Others	292 (17.2)	133 (16.2)	93 (16.3)	66 (22.0)	
**Clinical outcomes**					
28-day mortality, %	222 (13.1)	129 (15.7)	56 (9.8)	37 (12.3)	0.006
Length of ICU stay, day	16 (9–28)	16 (9–28)	16 (9–28)	14 (8–27)	0.04
**New receipt of organ support therapy within the first 7 days after enrollment**					
Mechanical ventilation	177 (10.4)	82 (10.2)	65 (11.3)	28 (9.3)	0.620
Renal replacement therapy	109 (6.4)	72 (8.7)	25 (4.3)	12 (4.0)	0.001
Vasoactive agents	153 (9.0)	91 (11.0)	39 (6.8)	23 (7.6)	0.016

Data are presented as n (%) or median (interquartile range, IQR).

BMI, Body Mass Index; APACHE II, Acute Physiology and Chronic Health Evaluation II; SOFA, Sequential Organ Failure Assessment; mNUTRIC, modified Nutrition Risk in the Critically ill; AGI, Acute Gastrointestinal Injure.

### Nutrition therapy

**FIGURE 2:**
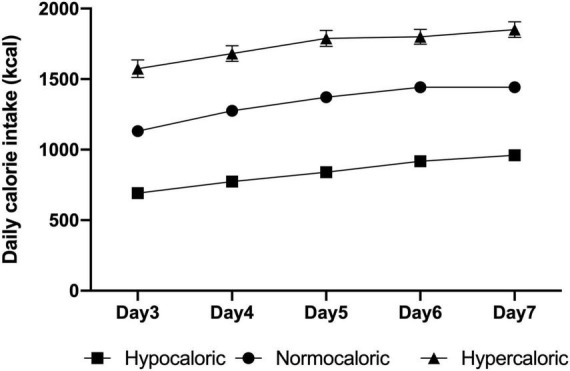
Daily calorie intake from days 3 to 7 for each group.

Data regarding nutrition therapy are shown in Table 2. The mean number of evaluable nutrition days was 4.49 days during the study period. The mean time of EN initiation was 2.17 days from study enrollment. There was no significance in prokinetics use among the three groups. The daily calories from days 3 to 7 after enrollment are shown in Figure 2. Overall, study patients received mean energy of 1,155.4 ± 456.5 kcal/day accounting for 73.8% of their estimated energy requirement. The mean protein intake was 45.3 ± 18.7 g/day.

**TABLE 2:** Nutrition therapy among three different categories of energy adequacy.

	Hypocaloric (*n* = 823)	Normocaloric (*n* = 571)	Hypercaloric (*n* = 300)	*P*-value
Number of evaluable nutrition days	4.28 ± 1.17	4.6 ± 0.82	4.71 ± 0.84	<0.001
**Nutrition process within the first 7 days after ICU admission**				
Time to first feeding,^a^ day	2.55 ± 1.52	1.86 ± 1.14	1.72 ± 1.10	<0.001
Initiation of EN within 48 h	494 (60.0)	459 (80.3)	257 (85.6)	<0.001
Total EN, %	683 (83.0)	500 (87.6)	251 (83.7)	0.058
Total PN, %	37 (4.5)	12 (2.1)	8 (2.7)	0.039
Patients receiving prokinetics, %	168 (20.4)	124 (21.7)	57 (19)	0.631
**Mean nutrition support days within the first 7 days after ICU admission, day**				
EN alone	4.65 ± 2.23	5.59 ± 1.85	5.58 ± 2.09	<0.001
PN alone	0.3 ± 0.95	0.15 ± 0.71	0.13 ± 0.65	<0.001
EN + PN	0.19 ± 0.69	0.21 ± 0.74	0.39 ± 1.14	0.124
**Mean energy and protein intake from day 3 to day 7**				
Energy adequacy, %	48.93 ± 15.36	85.14 ± 8.78	120.33 ± 19.61	<0.001
Energy intake, kcal/day	819.95 ± 276.72	1220.53 ± 234.28	1742.35 ± 401.49	<0.001
Protein intake, g/day	32.94 ± 12.28	52.48 ± 12.15	65.69 ± 18.65	<0.001

Data are presented as n (%) or mean ± SD.

EN, Enteral Nutrition; PN, Parenteral Nutrition; ICU, Intensive Care Unit.

^a^First feeding denotes the first time to receive EN or PN.

### Association between caloric adequacy and clinical outcomes

After the multicollinearity analysis for coprimary outcomes, the mNUTRIC score and protein intake were removed from the adjusted models given to its collinearity with SOFA score and calorie intake, respectively. After performing a univariate Cox analysis for coprimary outcomes, factors of clinical importance or those with a *p-*value of < 0.1 were then taken into the multivariate model as potential confounders. In terms of the 28-day mortality, the variables, such as age, gender, BMI, initiation of enteral nutrition within 48 h, mean parental nutrition intake from days 3 to 7, the number of evaluable nutrition days, SOFA score, and the number of comorbidities were entered into a multivariable model. For the outcome of time to discharge alive from the ICU, the covariates include age, gender, BMI, initiation of enteral nutrition within 48 h, the mean parental nutrition intake from days 3 to 7, and SOFA score. The relationships of caloric adequacy with 28-day mortality and time to discharge alive from the ICU are presented in Table 3. Compared with normocaloric feeding, hypocaloric feeding significantly increased the risk of 28-day mortality (hazard ratio [*HR*] = 1.590, 95% *CI*: 1.162–2.176, *p* = 0.004), while hypercaloric feeding did not (*HR* = 1.394, 95% *CI*: 0.920–2.112, *p* = 0.117). The association remains valid after controlling for potential confounders (age, gender, BMI, the number of evaluable nutrition days, initiation of enteral nutrition within 48 h, the mean parental nutrition intake from days 3 to 7, SOFA score, and the number of comorbidities), with an adjusted *HR* of 1.596 (95% *CI*: 1.150–2.215, *p* = 0.005). The caloric adequacy was not associated with time to discharge alive from the ICU in the unadjusted and the adjusted models.

**TABLE 3:** Relationship of energy delivery with coprimary outcomes.

	Normocaloric	Hypocaloric		Hypercaloric	
		Hazard ratio (95% CI)	*P*-value	Hazard ratio (95% CI)	*P*-value
**28-day mortality**					
Unadjusted analysis	Reference	1.590 (1.162–2.176)	0.004	1.394 (0.920–2.112)	0.117
Adjusted model^a^	Reference	1.596 (1.150–2.215)	0.005	1.249 (0.815–1.195)	0.307
**Time to discharge alive from the ICU**					
Unadjusted analysis	Reference	0.988 (0.858–1.137)	0.861	1.192 (0.998–1.423)	0.053
Adjusted model^b^	Reference	0.992 (0.856–1.150)	0.920	1.188 (0.989–1.426)	0.065

^a^Adjusted for age, gender, BMI, the number of evaluable nutrition days, initiation of enteral nutrition within 48 h, the mean parental nutrition intake from days 3 to 7, SOFA score, and the number of co-morbidities.

^b^Adjusted for age, gender, BMI, initiation of enteral nutrition within 48 h, the mean parental nutrition intake from days 3 to 7, and SOFA score.

**FIGURE 3:**
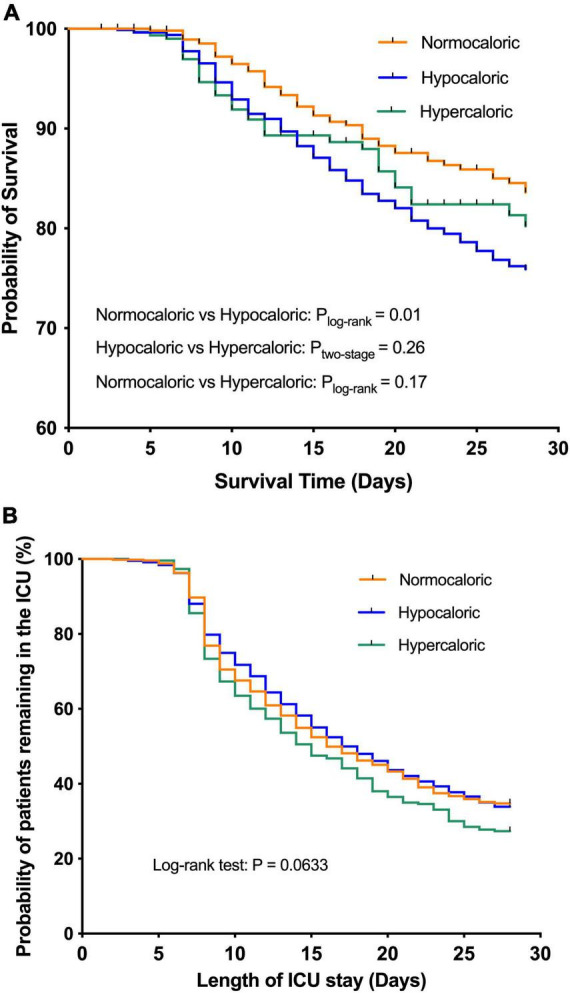
The Kaplan–Meier survival curves for the association of caloric adequacy with coprimary outcomes. **(A)** 28-day mortality. **(B)** Time to discharge alive from intensive care unit (ICU).

The Kaplan–Meier survival curves for the association of caloric adequacy with coprimary outcomes are shown in Figure 3. In terms of the 28-day mortality, the hypocaloric group was associated with a poor outcome compared with the normocaloric group (Figure 3A, *p*_two–stage_ = 0.01), while other comparisons did not differ. Meanwhile, there was no significant difference in the time to discharge alive from the ICU among the three groups (Figure 3B, *p*_log–rank_ = 0.0633).

## Discussion

Our study found that hypocaloric feeding (below 70% of energy requirement estimated by a weight-based equation) was associated with increased 28-day mortality, but caloric adequacy was not associated with time to discharge alive from the ICU in the unadjusted and adjusted models.

The optimal calorie intake remains controversial since many studies have shown conflicting results regarding the impact of different caloric adequacy on outcomes in critically ill patients. Several observational studies demonstrated that early target reaching is beneficial (24–26), whereas others reported that near-target caloric intake is associated with adverse outcomes (27, 28) and suggested hypocaloric nutrition (29, 30). Two randomized clinical trials (RCTs) found that low-dose caloric feeding during the first few days of ICU admission improved patient outcomes (5, 28), while others failed to detect a difference between underfeeding and full-energy feeding (31, 32). The heterogeneity in the study populations, different methods for resting energy expenditure (REE) measurements, and other clinical confounders, such as protein intake may account for these disputable conclusions.

The time range we selected in this study is based on the 2019 ESPEN guidelines (9), which divide the acute phase of critical illness into an early period (days 1–2) and a late period (days 3–7) and recommend progressively providing energy to cover at least 70% of the needs within days 3–7 (8). This definition is based on the pathophysiology of critical illness and previous studies. To make our results comparable with previous major studies and guidelines, we chose the same time range for this study. Our results showed that energy delivery below 70% of the energy requirement was associated with increased mortality, which is in line with Zusman et al.’s study (8). However, there was no difference between the hypercaloric feeding and the normocaloric feeding groups. A possible explanation for that is the different methods adopted for energy requirement estimation (IC vs. equation). According to the guidelines, IC is recommended as the optimum method for determining energy requirements among critically ill patients (9, 11). It can facilitate personalized, targeted nutrition therapy for critically ill patients (33). However, IC equipment is not readily accessible everywhere due to its high cost and technical clumsiness (10, 34, 35). Alternatively, the weight-based predictive equation, also recommended by the ASPEN guidelines (11), was widely used to estimate energy requirements because of its simplicity (10), although predictive equations might result in inaccurate energy requirement estimation (36). Previous studies showed that the weight-based predictive equation (25 kcal/kg/day) could provide a relatively accurate estimation of energy requirement (37, 38).

For nutrition therapy in critically ill patients, several observational studies have shown that adequate protein intake might outweigh calorie intake (39, 40). Two large-scale RCTs also confirmed the same (32, 41). The PERMIT trial compared permissive underfeeding (40–60% of caloric target) with standard enteral (70–100% of caloric target) while ensuring similar protein intake by supplements and found no differences in outcomes (32). Similarly, the TARGET trial failed to find a difference in 90-day survival between hypocaloric (69 ± 18% of calculated caloric needs) and eucaloric feeding (103 ± 27% of calculated caloric needs) when the protein intake was kept equal (41). However, in this study, protein intake could not be independently added for adjustment due to its collinearity with calorie intake. That is because “calorie intake (kcal/day)” and “protein intake (g/day)” usually have an inherent relationship in the commercially available enteral or parenteral formulas because both of them are regulated based on the concept of “Non-Protein Calorie: Nitrogen Ratio.”

This study has several strengths and limitations. First, the nutritional data in the analysis were prospectively collected during a large-scale RCT conducted in 90 ICUs, implicating good generalizability of the findings. Second, to minimize the bias, we did not calculate the energy and protein delivery when patients received oral intake. However, due to the nature of this study (a *post hoc* analysis), a causal relationship cannot be inferred. Third, since most of the study subjects did not undergo indirect calorimetry (IC) to evaluate energy expenditure, the generalizability of our findings was unknown in patients using IC to determine their energy requirements.

## Conclusion

This study showed that energy delivery below 70% of energy requirement during days 3–7 of critical illness is associated with increased 28-day mortality when using the weight-based equation to estimate the energy requirement.

## Data availability statement

The datasets used and analyzed during the current study are available from the corresponding authors on reasonable request.

## Ethics statement

The studies involving human participants were reviewed and approved by the Ethics Committee of Jinling Hospital. The patients/participants provided their written informed consent to participate in this study.

## Author contributions

LK, ZT, ZJ, and WL: conceptualization. XJ, ZL, and YiL: data curation. CL: formal analysis. RY, YXL, ML, YaL, and WC: methodology. CW, XY, and LG: resources. WL: supervision. JL and LK: writing – original draft. CL, XJ, and YXL: writing – review, and editing. All authors contributed to the article and approved the submitted version.
