# Supplementary material for: Association between caloric adequacy and short-term clinical outcomes in critically ill patients using a weight-based equation: Secondary analysis of a cluster-randomized controlled trial
Source: Front Nutr. 2022 Sep 2;9:902986. doi: 10.3389/fnut.2022.902986 (PMC9478102; doi:10.3389/fnut.2022.902986)
Supplement: Supplementary file 1 [file Data_Sheet_1.pdf]

Additional information to

Association between calorie adequacy and short-term clinical outcomes in critically ill patients using a weight-based equation: secondary analysis of a cluster-randomized controlled trial

Cheng Lv [1,#], Xingwei Jiang [2,#], Zirui Liu [1], Jiajia Lin [1], Yi Long [3], Cuili Wu [4], Xianghong Ye [4], Ruiling Ye [3], Yuxiu Liu [1], Man Liu [1], Yang Liu [1], Wensong Chen [5], Lin Gao [1], Zhihui Tong [1], Lu Ke [1,6,*], Zhengying Jiang [3,*], Weiqin Li [1,6,*] for the Chinese Critical Care Nutrition Trials Group (CCCNTG)

1. Department of Critical Care Medicine, Jinling Hospital, Medical School of Nanjing University, Nanjing, 210002, PR China.
2. Department of Critical Care Medicine, Jinling Hospital, Medical School of Southeast University, Nanjing, China.
3. Department of Critical Care Medicine, Chongqing University Cancer Hospital, Chongqing, 400030, PR China.
4. Department of General Surgery, Jinling Hospital, Medical School of Nanjing University, Nanjing, 210002, PR China.
5. Department of Biostatistics, School of Public Health, Nanjing Medical University, Nanjing, China.
6. National Institute of Healthcare Data Science, Nanjing University, Nanjing, China.

# Cheng Lv and Xingwei Jiang have contributed equally to this work.

CONTENTS

Table S1. Univariate Cox analysis for 28-day mortality……………………………………………………………………………Page 2
Table S2. Univariate Cox analysis for time to discharge alive from the ICU within 28 days after enrollment………………………………Page 3
Table S3. Subgroup analyses for energy delivery and coprimary outcomes in patients with different malnutrition risks…………………….Page 4
Figure S1. Restricted cubic spline for 28-day mortality……………………………………………………………………… Page 5
Figure S2. Restricted cubic spline for time to discharge alive from ICU……………………………………………………………Page 6

Table S1. Univariate Cox analysis for 28-day mortality.

	Hazard ratio	95% CI	P value
Age	1.010	1.003-1.018	0.008
Male	0.907	0.689-1.195	0.487
BMI	0.919	0.880-0.959	<0.001
APACHE II	1.055	1.036-1.074	<0.001
SOFA	1.073	1.036-1.111	<0.001
Number of co-morbidities	1.102	1.035-1.173	0.002
mNUTRIC score	1.204	1.126-1.288	<0.001
Number of Evaluable nutrition days	0.887	0.791-0.995	0.041
EN started within 48 hours after enrollment	0.815	0.616-1.079	0.153
Mean PN intake from day3 to day7	1.000	0.971-1.030	0.990
**AGI score**			
AGI I	reference		
AGI II	1.096	0.791-1.517	0.582
AGI III-IV	1.148	0.696-1.893	0.589

BMI, Body Mass Index; APACHE II, Acute Physiology and Chronic Health Evaluation II; SOFA, Sequential Organ Failure Assessment; mNUTRIC, modified Nutrition Risk in the Critically ill; EN, enteral nutrition; PN, parenteral nutrition; AGI, Acute Gastrointestinal Injure.

Table S2. Univariate Cox analysis for time to discharge alive from the ICU after enrollment.

	Hazard ratio	95% CI	P value
Age	0.996	0.992-0.999	0.016
Male	0.981	0.859-1.121	0.782
BMI	1.002	0.983-1.021	0.858
APACHE II	0.987	0.978-0.997	0.008
SOFA	0.975	0.958-0.993	0.007
Number of co-morbidities	1.006	0.972-1.042	0.730
mNUTRIC score	0.943	0.913-0.974	<0.001
Number of Evaluable nutrition days	0.962	0.906-1.021	0.202
EN started within 48 hours after enrollment	0.982	0.855-1.128	0.800
Mean PN intake from day3 to day7	1.004	0.990-1.017	0.610
**AGI score**			
AGI I	reference		
AGI II	0.911	0.774-1.071	0.259
AGI III-IV	0.956	0.744-1.227	0.723

BMI, Body Mass Index; APACHE II, Acute Physiology and Chronic Health Evaluation II; SOFA, Sequential Organ Failure Assessment; mNUTRIC, modified Nutrition Risk in the Critically ill; AGI, Acute Gastrointestinal Injure.

Table S3. Subgroup analyses for relationship between energy delivery and coprimary outcomes in patients with different malnutrition risks.

	Normocaloric	Hypocaloric		Hypercaloric	
		Hazard ratio (95% CI)	P value	Hazard ratio (95% CI)	P value
Low risk of malnutrition (mNUTRIC≤4)					
28-day mortality	reference	2.019 (1.219-3.342)	0.006	1.125 (0.542-2.334)	0.752
Time to discharge alive from the ICU	reference	1.082 (0.902-1.299)	0.394	1.212 (0.967-1.520)	0.096
High risk of malnutrition (mNUTRIC>4)					
28-day mortality	reference	1.281 (0.857-1.913)	0.227	1.542 (0.928-2.562)	0.095
Time to discharge alive from the ICU	reference	0.882 (0.706-1.101)	0.268	1.160 (0.870-1.546)	0.312

Figure S1. Restricted cubic spline for 28-day mortality*

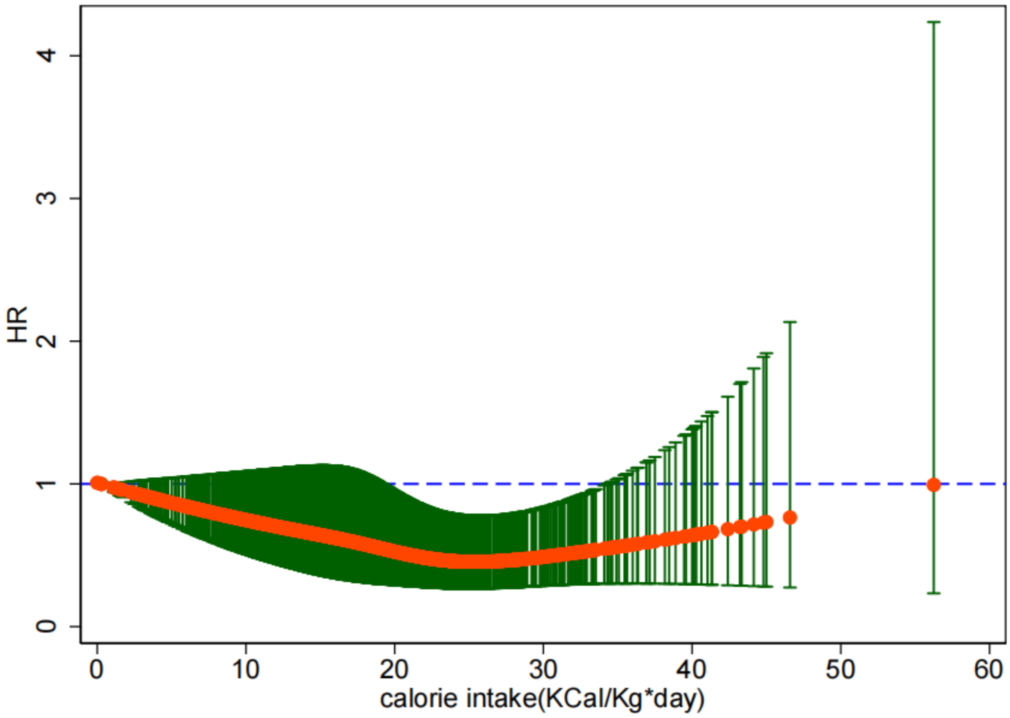

*Adjusted for age, gender, BMI, the number of evaluable nutrition days, initiation of enteral nutrition within 48 hours, mean parental nutrition intake from day3 to day7, SOFA score and number of co-morbidities.

Figure S2. Restricted cubic spline for time to discharge alive from ICU*

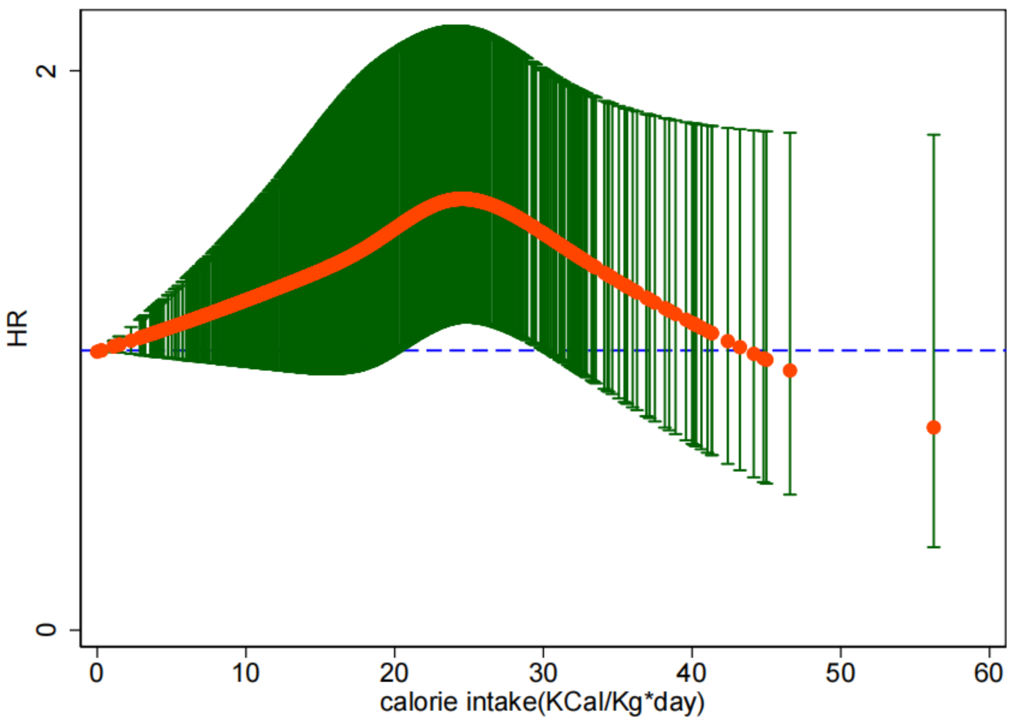

*Adjusted for age, gender, BMI, initiation of enteral nutrition within 48h,SOFA score.
